# Estimation of intake and quantification of hemoglobin adducts of acrylamide in adolescents in Sweden

**DOI:** 10.3389/fnut.2024.1371612

**Published:** 2024-06-03

**Authors:** Efstathios Vryonidis, Margareta Törnqvist, Sanna Lignell, Johan Rosén, Jenny Aasa

**Affiliations:** ^1^Department of Environmental Science, Stockholm University, Stockholm, Sweden; ^2^Division of Risk and Benefit Assessment, Swedish Food Agency, Uppsala, Sweden; ^3^Division of Laboratory Investigation and Analysis, Swedish Food Agency, Uppsala, Sweden

**Keywords:** chemical exposure, hemoglobin adducts, acrylamide, dietary exposure, adolescents

## Abstract

Blood samples (*n* = 600) from participants in the Swedish dietary survey Riksmaten Adolescents 2016–17 were analyzed with respect to hemoglobin (Hb) adducts from acrylamide (AA) and its metabolite glycidamide (GA) as biomarkers of internal dose/exposure. The results are presented from statistical analyses of food consumption data (2-day dietary recall and questionnaires) and measured Hb adduct levels. The estimated exposure as well as consumption data were examined in relation to non-dietary factors such as sex, age (group medians of 12, 15, and 18 years), place of residence (urban/rural), smoking status, and parental education level. The median AA adduct level was estimated to be 34 pmol/g Hb (range 14–225). No significant difference was found for place of residence, parental education, sex, or age. A significant difference was found between the median adduct levels of daily smokers (*n* = 8) and never smokers (*n* = 323) in the older age groups, but not between occasional smokers (*n* = 47) and never smokers. The median differences between daily smokers and never smokers were 76, 40, and 128 pmol/g Hb for AA, GA, and AA + GA, respectively. The median AA intake for the whole group of adolescents, as estimated from dietary recall data combined with reported concentrations in food, was 0.40 μg/kg bw/day. The corresponding median intake estimated from measured Hb adduct levels of AA was 0.20 μg/kg bw/day. A significant, although low, positive Spearman correlation was found between the two intake estimates (*p*-value = 8 × 10^−3^; ρ = 0.11). From the estimated intake of AA from food frequency questionnaires, significance was found for the 15-year-old children with higher AA adduct levels observed at higher consumption frequencies of fried potatoes/French fries. AA is considered a genotoxic carcinogen. For the estimated intake of AA for any age group and method (dietary recall or AA adduct), both a calculated margin of exposure as well as lifetime quantitative cancer risk estimates indicate health concern. A future study on food consumption designed with respect to AA exposure would provide a better understanding of the correlation between consumption and exposure and should give a more reliable estimate of the contribution of dietary AA to the overall cancer risk.

## 1 Introduction

In 2016–2017, the Swedish Food Agency conducted a national dietary survey to collect information on food consumption among Swedish adolescents, Riksmaten Adolescents 2016–17 (1, 2). The data have been used to calculate nutrient and energy intakes, as well as the exposure to several unwanted compounds from food (3). In parallel with the dietary survey, blood and urine samples were collected from a subgroup of participants for biomonitoring of several parameters. Knowledge about internal exposures to unwanted compounds enables improvement of health risk assessments and has been generated from samples from this cohort for several chemical compounds (4–7).

One compound that we are commonly exposed to from food is acrylamide (AA, 2-propenamide, CAS No. 79–06-01), which is classified as probably carcinogenic to humans (Group 2A) by the International Agency for Research on Cancer (8), and which also could cause other toxic effects (9). It is also known as an industrial chemical used in the production of polyacrylamides, primarily used as flocculants in drinking water treatment plants (9). In food, AA is formed during the preparation of carbohydrate-rich foods, i.e., potatoes (French fries, crisps), bread, biscuits, and coffee, at temperatures above 120°C and at low moisture (9, 10). This occurs through a Maillard reaction (“browning of food”), particularly between reducing sugars and the amino acid asparagine (11). AA is also present in cigarette smoke (12), and studies show that regular smokers usually have several times higher exposure to AA compared to non-smoking individuals, as measured by hemoglobin (Hb) adducts (13). Furthermore, it has been indicated that AA could be formed from endogenous processes, which thus might be an additional source of exposure (14). AA is metabolized *in vivo* to glycidamide (GA, 2,3-epoxypropanamide, CAS No. 5694-00-8) via the cytochrome P450 2E1 enzyme (15). GA has been shown to be genotoxic and carcinogenic in animal studies (9).

Due to the toxic properties of AA and GA, it is important to monitor the internal doses of these compounds and measure the exposure to AA in the general population. Biomonitoring of AA and GA is commonly performed in blood or urine. Both AA and GA are electrophilic and thus could bind covalently with nucleophilic sites in biomacromolecules and form stable reaction products (adducts), which could be used for biomonitoring. One such reaction is between the compounds and the N-terminal valines in the Hb chains, creating Hb adducts, which are monitored in blood samples (16, 17). In urine, AA and GA can be monitored as their mercapturic acid metabolites, and about 10–20% of AA is found to be metabolized to GA in humans (18–20).

In the present study, the internal dose (concentration over time) of AA, measured through Hb adducts, as well as the dietary exposure to AA are studied in individuals from the Riksmaten Adolescents dietary survey. This article summarizes the results from the analyses of AA and GA (as Hb adducts) in blood samples from a subset of these individuals (*n* = 600). For the adolescents, associations of Hb adduct levels to non-dietary factors, namely age (school grade), type of residence (urban/rural), parental education, smoking status, and sex, were investigated. We evaluated associations between Hb adduct levels and consumption of foods known to contain AA, using dietary recall data combined with concentrations in food to estimate the intake of AA. The AA intake estimated from dietary recall data, was compared with AA intake estimated from AA Hb adduct levels. Moreover, the relationship between Hb adduct levels and AA intake data estimated from web questionnaires on food consumption over the previous 12 months was investigated.

## 2 Materials and methods

### 2.1 Chemicals

The internal standards of the adduct analytes (fluorescein thiohydantoins, FTHs) were synthesized in-house and characterized previously (21). Peptides of the Val adduct of acrylamide [*N*-(2-carbamoyl-ethyl)-Val-Leu-anilide; CAS: 282725–67-1] and glycidamide (N-[(RS)-2-carbamoyl-2-hydroxy-ethyl]-Val-Leu-anilide; CAS: 1926163–77-0) were purchased from Bachem, Switzerland. Cyanoacetic acid (CAS: 372–09-8), sodium lauryl sulfate (CAS: 151–21-3), sodium phosphate monobasic monohydrate (CAS: 10049–21-5), and sodium phosphate dibasic (CAS: 7558-79-4) were purchased from Sigma-Aldrich. Ammonia solution 25% (CAS: 1336-21-6) and sodium chloride (CAS: 7647-14-5) were purchased from Saveen & Werner, Sweden. Fluorescein isothiocyanate (FITC) isomer I (CAS: 3326-32-7) was obtained via Chemtronica, Sweden. Acetonitrile, water, and isopropanol of HPLC quality (Honeywell) were purchased from Saveen & Werner, Sweden.

### 2.2 Study subjects and sample collection

The present investigation was performed with blood samples earlier collected from participants of a subgroup of Riksmaten Adolescents 2016–17, a national dietary survey among adolescents (10–21 years) conducted by the Swedish Food Agency (1, 22). In brief, 2,377 students from 62 schools from all parts of Sweden were invited to participate in the biomonitoring part of the study. In total, 1,105 students in fifth grade (median age 12 years), eighth grade (median age 15 years), and in the second grade of high school (median age 18 years) provided complete biological samples and complete dietary information. Ethical approval for the survey was obtained from the Regional Ethical Review Board in Uppsala (No. 2015/190). Written informed consent was obtained from all participants and from the legal guardians of individuals younger than 16 years. The blood samples were stored at −80°C at the Swedish Food Agency after the collection. A fraction of 750 μL of whole blood per sample was further transferred to plastic test tubes and stored at −80°C before chemical analysis of Hb adducts of AA and GA.

In the present study, approximately 200 blood samples per age group (per school grade), i.e., 600 samples in total, equally distributed between sex, were selected for analysis of AA and GA (as Hb adducts) from the biomonitoring part of the Riksmaten Adolescents study. The rationale for the selection was to enable an equal distribution between sex and grade in school and an equal distribution between the geographical locations of the schools in Sweden (from north to south). The characteristics of the participants are included in Table 1. The two oldest age groups were asked about smoking habits, but the number of smoked cigarettes per day was not given (Table 1).

**Table 1:** Characteristics of the adolescents in Riksmaten Adolescents 2016–17 who provided blood samples that were used for acrylamide analysis (*n* = 600).

	Fifth grade (*n* = 201)	Eighth grade (*n* = 200)	Second grade of high school (*n* = 199)
Female (*n*)	100	100	100
Male (*n*)	101	100	99
**Smoking status^a^ (*n*):**			
Daily smokers	0	0	8
Occasional smokers	0	10	40
Never smokers	0	181	142
Quitted smoking	0	3	6
Did not want to answer	0	6	3
Not Asked	201	0	0
Median age (years) [min–max], P05–P95	11.5 [10.8–13.1], 11.0–12.3	14.6 [13.3–15.7], 13.9–15.1	17.8 [16.8–21.0], 17.0–19.2
Median weight (kg) [min–max], P05–P95	43.3 [25.9–81.6], 30.8–60.5	57.5 [37.7–107.1], 42.6–79.1	69.2 [45.1–124.4], 51.0–92.9
Municipality (n) urban/rural	142/59	150/50	122/77
**Parental education level (*n*):**			
Elementary school	9	6	10
High school	62	56	75
University	125	131	102
No answer	5	7	12

^a^No information is available for any other tobacco use.

### 2.3 Assessment of diet

Diet was assessed using a validated web-based 24-h dietary recall method, RiksmatenFlexDiet, developed by the Swedish Food Agency (23, 1). With this method, the participants recorded their consumption of food and beverages retrospectively on two non-consecutive days. The second day was randomly assigned to occur 2–7 days after the first recall day. RiksmatenFlexDiet included 778 main food items that were available to choose from. In the second step of the registration, it was possible to add information on ingredients, e.g., type of meat in a stew, type of berries in jam, which resulted in more than 2,300 food items in total. The method fulfils the recommendation from the European Food Safety Auhority (EFSA) to use a 24-h recall method including two non-consecutive days for the collection of national food consumption data (24). As a complement to the dietary recall data, web questionnaires were used to estimate the consumption frequencies of selected foods over the previous 12 months. In the present study, we used data collected on consumption frequencies of AA-rich foods, i.e., fried potatoes/French fries, bread, and crisps.

### 2.4 Estimation of acrylamide intake from dietary recall

Based on the dietary recall data (2 days of registration), the mean consumption (g/day) of food groups relevant for AA intake (i.e., bakery and cakes, soft bread, cereals, chocolate/cocoa products, coffee, crispbread, crisps, dried fruit, French fries, and fried potatoes) was calculated for the participating adolescents. The daily intake of AA was further estimated by combining the consumption data for the individuals with data on AA levels in the different food groups, either from analyses by the Swedish Food Agency in 2022 [*cf.* for the method used] or from reported data in the scientific opinion on AA by EFSA (9). For the AA concentration in different food items used for the calculations, see Table 2.

**Table 2:** Acrylamide mean concentrations (μg/kg) in different food categories used for calculations of daily intake.

Food category	Reference	Subcategory	Acrylamide concentration in subcategory (μg/kg)	Mean acrylamide concentration in food category (μg/kg)
Bakery and cakes	(9)	–	–	271 (*n* = 1735)^b^
Bread (soft)	SFA, 2022	Rye/wheat	22.7 (*n* = 9)^a^	38 (*n* = 18)^a^
		Whole grain, dark bread	52.8 (*n* = 9)^a^	
Breakfast cereals	SFA, 2022	Corn	28.0 (*n* = 6)^a^	76 (*n* = 19)^a^
		Wheat/rice/oat	128 (*n* = 7)^a^	
		Crunchy muesli	71.2 (*n* = 6)^a^	
Chocolate/cocoa products	(9)	–	–	73 (*n* = 31)^b^
Coffee	SFA, 2022	Dark roast, major brands	10.7^c^ (*n* = 6)^a^	13^c^ (*n* = 22)^a^
		Medium roast, major brands	13.3^c^ (*n* = 6)^a^	
		Medium roast, smaller brands	14.5^c^ (*n* = 10)^a^	
Crispbread	SFA, 2022	Fermented rye bread	125 (*n* = 6) ^a^	138 (*n* = 18)^a^
		Non-fermented rye bread	220 (*n* = 5)^a^	
		Wheat/oat	70.0 (*n* = 7)^a^	
Crisps	SFA, 2022	Main domestic producers	432 (*n* = 12)^a^	341 (*n* = 36)^a^
		Private label	353 (*n* = 12)^a^	
		Others	237 (*n* = 12)^a^	
Dried fruit	(9)	–	–	89 (*n* = 18)^b^
French fries and fried potatoes	SFA, 2022	Major fast-food chains	223 (*n* = 6)^a^	300 (*n* = 18)^a^
		Street food	200 (*n* = 6)^a^	
		Restaurants	476 (*n* = 6)^a^	

Occurrence data are either derived from analyses of food items at the Swedish Food Agency during 2022 (unpublished data, denoted as SFA, 2022 in the table) or from reported data used for exposure assessment in Appendix D of the scientific opinion on acrylamide by the (9). ^a^Regarding this data (i.e., from the Swedish Food Agency), “n” denotes the total number of food samples of different brands or recipes that were homogenized separately and then pooled in equal amounts to one composite sample per subcategory. Each composite sample was thereafter analyzed at least twice at different days and by different operators. The analytical method used (25) has been subjected to collaborative trial validation (26, 27). ^b^Regarding the EFSA data, “n” denotes the total number of samples per food category reported to EFSA from EU countries. ^c^Estimated level in ready-to-drink coffee from occurrence level in coffee powder divided by the factor 18.6 (dilution factor based on a recipe where 50 g of coffee powder is used with 1 L of water when brewing and where a higher relative amount of water than acrylamide is stuck to the filter paper, as observed from measurements).

### 2.5 Measurement of Hb adduct levels and Hb concentration

#### 2.5.1 Analysis of Hb adducts by the FIRE procedure

Samples were randomly divided into batches of up to 87 samples. Two samples were created from each blood sample, one to measure the adduct levels of AA and GA and one to determine the Hb concentration in the sample.

The FIRE procedure was used for the analysis of the N-terminal Val adducts in Hb as FTHs by LC–MS (21). In the current study, some modifications and optimizations were made to allow for the use of 96 deep well plates (DWP). To 150 μL whole blood samples, 20 μL 0.1 μM of isotopically substituted internal standards of AA (d7) Val FTH and GA (d7) Val FTH were added. Thereafter, 100 μL of a solution containing AA (d₃)-modified Hb in 0.15 M phosphate buffer (disodium hydrogen phosphate:sodium dihydrogen phosphate; 9:1; pH ca 7.8) was added. Additionally, 100 μL of water was added to the samples before mixing by shaking at 1500 rpm for 1 min. The samples were derivatized for 16 h at 750 rpm and 37°C with 3 mg fluorescein isothiocyanate (FITC) in 18 μL dimethyl sulphoxide.

After derivatization, 50 μL methanol was added to the samples, which were then mixed at 1000 rpm for 1 min. Thereafter, the samples were transferred to a protein precipitation filter plate (Isolute PPT+, Biotage, Sweden) pre-loaded with acetonitrile as crash solvent. The filtrate of the samples was collected in a 2 mL DWP, and to that, 15 μL of 1 M NH₄OH was added, followed by loading on Oasis MAX 96-well SPE plate (Waters, Sweden) that was preconditioned with 0.01 M NH₄OH. The samples were then washed with 1 mL ACN, 1 mL water, and 1 mL 0.5% cyanoacetic acid in water. To elute the samples, 0.8 mL 0.25% cyanoacetic acid in 80% ACN was used, and the samples were evaporated to dryness overnight in a vacuum centrifuge at room temperature. Prior to LC–MS analysis, the samples were reconstituted in 100 μL 40% acetonitrile in water.

#### 2.5.2 LC–MS analysis

Analysis of the samples was performed on a Waters Acquity H-class UPLC coupled to a Xevo TQS-μ tandem-MS instrument (Waters Corporation, Milford, MA, USA). The analytical column used was an ACE Excel UHPLC C18-PFP column (100 mm × 2.1 mm, 1.7 μm). Elution proceeded at 0.35 mL/min, with the column temperature kept at 40°C. The mobile phases used were composed of: (A) 10% acetonitrile in water with 0.1% formic acid; and (B) 90% acetonitrile in water with 0.1% formic acid. The gradient program used is given in Supplementary Figure S1.

The MS was operated in ESI-positive mode. Capillary voltage was set at 1.00 kV, source temperature at 130°C, desolvation temperature at 600°C, cone gas flow at 60 L/h, and desolvation gas flow at 1200 L/h. The instrument was operated in multiple reaction monitoring mode, and 2–3 transitions were used per precursor ion with varying cone voltage and collision energy (see Supplementary Table S1 for a list of the monitored transitions, and Supplementary Figure S2 for the presumed parent ions of the analytes).

#### 2.5.3 Quality control and calibration curve samples

Each batch contained triplicates of quality control (QC) blood samples at three different levels of the AA adduct (blood of a non-smoker, blood of a smoker, and blood of a non-smoker spiked with erythrocytes incubated with AA). The QC samples of each batch were used to correct for batch effect by normalizing the response of each batch to that of the calibration curve batch (see Supplementary material, section 2 for the formula used). The calibration curve batch contained five QC replicates of each level.

The intraday precision of the analysis, measured as the mean coefficient of variation per batch of the uncorrected relative response of the AA analyte on the three QC levels, was, on average, 13%. The interday precision of the analysis, measured as the coefficient of variation of the uncorrected relative response of the AA analyte, was measured to be: QC low = 21%; QC medium = 17%; QC high = 21%.

The instrumental limit of detection (LOD) was calculated to be 0.3 fmol on the column of AA-Val-FTH, based on the approach by Agilent Technologies, Inc. (28), determined from repeated injections of FTH standard for AA at a response level very close to LOD (S/N = 3.5–7.4). By assuming that the instrumental LOD is the same for the analysis of a blood sample with a Hb concentration of 140 mg/mL (the mean Hb concentration observed in samples), this LOD would equate to an adduct level of 0.5 pmol/g Hb. In the analysis of samples, the level of detection was set to 3 S/N, and the limit of quantification was set to 10 S/N.

For the calibration curve samples used for determining the adduct level in the samples, commercial blood from a non-smoker was spiked with peptides producing the AA-Val-FTH and GA-Val-FTH analytes upon derivatization with FITC. Namely, the peptides used were N-(2-carbamoyl-ethyl)-Val-Leu-anilide for AA-Val and N-[(RS)-2-carbamoyl-2-hydroxy-ethyl]-Val-Leu-anilide for GA-Val. Calibration curve samples were processed as the rest of the samples, with the exception that 20 μL less water was added in the initial dilution step to compensate for the volume of the 20 μL of spike peptide standard that was added. In total, seven levels + control were used for the calibration curve, with the amount of spike standard added being 3.7, 3.1, 2.4, 1.8, 1.2, 0.6, 0.1, and 0 pmol peptide per sample, and a triplicate spiked sample per level was made.

#### 2.5.4 Estimation of Hb concentration in samples

To determine the concentration of Hb in the samples, the sodium lauryl sulfate (SLS) method for measuring Hb concentration was used, introduced by Oshiro et al. (29), with modifications and adaptations to the 96-well plate format.

In short, an SLS stock solution was made by dissolving 3.0 g of SLS in 50 mL of water containing 1 mL of 1 M disodium hydrogen phosphate solution and 0.5 mL of 1 M sodium dihydrogen phosphate solution. The SLS stock solution was then diluted 100 times in water, and the resulting SLS working solution was used to dilute 10 μL of whole blood samples from the study 151 times in a 2 mL deep well plate. The SLS blood samples were then mixed, and 300 μL were transferred to a microplate, where the optical density of the samples was measured at 538 nm in a SpectraMax iD3 microplate reader (Molecular Devices, USA). To estimate the Hb concentration of the study samples, erythrocyte solutions of known Hb concentration were run at the same occasion. No correction was applied post-analysis because all SLS plates were processed and analyzed at the same occasion.

### 2.6 Calculation of acrylamide intake from Hb adduct levels

AA daily intake per kg bw (body weight) was estimated from measured Hb adduct levels by using earlier data on adduct increment per unit AA intake from a dietary intervention human study. Hb adduct levels reflect the internal dose in blood, in the meaning of “area under the concentration-time curve (AUC)” (in M × h) during the period of exposure (30). The internal dose can be calculated from the adduct level when the rate constant for adduct formation and the half-time of the adduct level are known. Adducts to Hb from exposure are formed over the lifetime of the erythrocytes (*t*_er_, *ca.* 126 days in humans; (31)) and disappear with Hb following the elimination of old erythrocytes. At constant exposure over *t*_er_ a steady-state adduct level is reached, which corresponds to daily adduct level increments at *ca.* 63 days, that is *t*_er_/2. In the present study, the daily internal dose of AA was calculated from the daily AA adduct level increment by using the rate constant for the formation of AA adduct to N-terminal valine in Hb (*k*_Val-AA_). We assume that the measured AA adduct level in adolescents is at an approximate steady-state level. The daily AA intake per kg bw was then calculated using data from an earlier human dietary intervention study (32, 33). The *k*_Val-AA_ measured in the present study also functions as a calibration to the internal dose from the previous data used for calculation of intake [required because that adduct data were obtained with another method/analytical instrument as described in detail by Vryonidis et al. (34)].

In the present study, the *k*_Val-AA_ was determined by using human blood samples incubated with AA, which were processed and analyzed in parallel to the cohort samples. Five different concentrations of AA (C_0_ = 0, 20, 60, 80, and 140 μM AA) were used in the incubation experiment in blood from a single individual, and incubation duration was 1 h. The preparation of these samples was described earlier (34).

The previous study, mentioned above, was a dietary intervention with adults (data from non-smokers), consuming either AA-rich food (10 individuals, 11 μg/kg bw/day) or food with very low levels of AA (9 individuals) for 4 days (32, 33). In that study, the AA intake was estimated from the occurrence of AA in pooled lunches and from food diaries combined with AA content in other food items and linked to internal doses of AA, calculated from Hb adduct levels. The calculated daily increase of adduct levels was 1.36 ± 0.36 pmol/g globin per μg AA/kg bw/day, which corresponded to an internal dose (AUC) of 212 nM × h AA per μg AA/kg bw/day (16, 33).

### 2.7 Calculations and statistical analysis

Statistical analyses were performed using the RStudio IDE (Version 2022.7.1.554). The normality of the data was investigated with Lilliefors, Shapiro–Wilk, and Anderson–Darling normality tests. To examine the probability differences in the data of the two groupings, the Mann–Whitney *U* test was used. Kruskal–Wallis tests were used for one-way analysis of variance in the data. Dunn’s test with Bonferroni correction was utilized as a *post-hoc* test following significant results from Kruskal–Wallis tests. To study the association in the data, Spearman rank correlation tests were performed.

## 3 Results and discussion

### 3.1 Hb adduct levels

#### 3.1.1 Characteristics of the cohort with regard to Hb adduct levels

The present study has been performed on a representative sample of Swedish adolescents. The characteristics of the included adolescents are shown in Table 1. The hemoglobin concentrations in the samples were measured to be 140 ± 14 g/mL (mean ± *SD*). The levels of the Hb adducts of AA, GA, and AA + GA for the different age groups are presented in Table 3.

**Table 3:** Levels of Hb adducts of acrylamide, glycidamide, and the sum of the adducts (AA–Hb + GA–Hb) among the adolescents in fifth grade (12 years), eighth grade (15 years), and second grade of high school (18 years).

		Acrylamide Hb adducts (pmol/g Hb)			Glycidamide Hb adducts (pmol/g Hb)			AA–Hb + GA–Hb (pmol/g Hb)		
Grade	Sex	Mean ± *SD* (detection ratio)	Median [min–max]	P05–P95	Mean ± *SD* (detection ratio)	Median [min–max]	P05–P95	Mean ± *SD* (detection ratio)	Median [min–max]	P05–P95
All grades (*n* = 600)	All	38 ± 18 (594/600)	34 [14–225]	20–65	55 ± 32 (452/600)	50 [9–453]	23–102	94 ± 48 (452/600)	85 [29–678]	48–169
	♀	38 ± 16 (298/300)	34 [15–156]	21–65	54 ± 28 (215/300)	50 [10–255]	23–102	93 ± 42 (215/300)	83 [36–410]	49–169
	♂	38 ± 19 (296/300)	34 [14–225]	20–67	55 ± 36 (237/300)	50 [9–453]	22–103	95 ± 53 (237/300)	87 [29–678]	47–166
Fifth grade (*n* = 201)	All	39 ± 13 (198/201)	37 [17–90]	22–62	59 ± 23 (159/201)	58 [19–154]	25–101	99 ± 32 (159/201)	96 [38–195]	54–164
	♀	39 ± 12 (98/100)	37 [17–74]	23–62	57 ± 23 (70/100)	56 [19–136]	23–92	96 ± 32 (70/100)	94 [38–174]	54–155
	♂	39 ± 13 (100/101)	37 [18–90]	22–60	62 ± 24 (89/101)	59 [22–154]	28–102	101 ± 32 (89/101)	96 [42–195]	58–165
Eighth grade (*n* = 200)	All	37 ± 19 (200/200)	33 [14–225]	20–65	51 ± 39 (151/200)	48 [10–453]	21–84	90 ± 58 (151/200)	81 [36–678]	47–161
	♂	35 ± 13 (100/100)	31 [15–103]	21–62	47 ± 17 (73/100)	47 [10–102]	22–70	83 ± 27 (73/100)	79 [36–169]	48–137
	♂	39 ± 24 (100/100)	34 [14–225]	20–73	56 ± 52 (78/100)	48 [15–453]	21–99	96 ± 76 (78/100)	82 [37–678]	47–164
Second grade of high school (*n* = 199)	All	38 ± 21 (196/199)	33 [14–156]	19–69	53 ± 32 (142/199)	47 [9–255]	20–116	93 ± 51 (142/199)	81 [29–410]	43–203
	♀	40 ± 22 (100/100)	33 [17–156]	20–68	58 ± 38 (72/100)	49 [17–255]	23–129	100 ± 59 (72/100)	83 [40–410]	51–211
	♂	37 ± 20 (96/99)	31 [14–129]	18–68	47 ± 24 (70/99)	43 [9–116]	18–101	86 ± 41 (70/99)	75 [29–245]	38–173

^♀^girls. ^♂^boys. *SD*, standard deviation.

A widespread exposure to AA was observed, with the median level of AA adducts being 34 pmol/g Hb (range 14–225). The quantification frequency in the samples for the GA analyte was lower (75%) than for AA (99%). This is to a large extent due to difficulties with the current state of the analytical method for the detection of the GA analyte (lower overall signal-to-noise ratio). The median ratio of GA to AA (GA/AA) adduct level (in participants with quantifiable levels of both GA and AA adducts) is 1.4 (range 0.4–5.0).

The observed adduct levels are comparable with levels observed in other studies of Hb adducts from AA in the general population [e.g., summarized by (13)]. According to earlier studies, GA adduct levels in non-smokers are expected to be somewhat higher than AA adduct levels. In accordance with the present result, e.g., Vikström et al. (16) found a median of 1.6 (range 0.6–6.7) for the GA/AA adduct level ratio (68 non-smoking adults). It should be noted that GA has higher reactivity with the N-terminal valine compared to AA per unit of dose (concentration over time) (33). Due to the observed fairly large inter-individual differences in the GA/AA adduct level ratio, the sum of AA and GA adduct levels has been discussed as a good measure of the total AA internal dose (35). Therefore, statistical analyses have been performed both on AA, GA, and AA + GA Hb adducts, as further discussed below. The inter-individual difference in GA/AA adduct level ratio is assumed to be due to difference in genotypes of metabolizing enzymes, primarily the CYP2E1 enzyme metabolizing AA to GA (36). In addition to the variation between individuals, Vikström et al. (16) also observed an intra-individual difference (up to 2-fold) in the adduct level ratio (11 non-smokers, repeated sampling over 20 months). From observations in other published studies, Vikström et al. discussed that the intra-individual difference would be due to the intake of specific components in food and of alcohol influencing the metabolism of AA to GA.

#### 3.1.2 Procedures for Hb adduct level measurement—improvements

The FIRE procedure was used to analyze AA adducts to N-terminal valine in Hb (17, 21). In the present study, an updated version of that procedure was used, where sample preparation is adapted to 96DWP and analysis is performed by UHPLC–MS. This variant has earlier been employed for AA adduct analysis (34). Furthermore, the Hb concentration was measured by SLS [a method by (29)], also with adaptation to 96DWP instead of the method used previously, where the measurement was performed on one sample at a time. These modifications have improved the method considerably with regard to time required for sample preparation and analysis, as discussed by Vryonidis (37). On the other hand, the present application uses less sample volume of blood than in previous applications (150 μL instead of 250 μL), which had some negative impact on the quantification frequency of GA adduct levels.

### 3.2 Acrylamide intake, food consumption pattern, and Hb adduct levels

#### 3.2.1 Acrylamide intake from dietary recall

AA intake was estimated from the mean daily consumption of selected food groups (bakery and cakes, soft bread, cereals, chocolate/cocoa products, coffee, crispbread, crisps, dried fruit, French fries, and fried potatoes) from the dietary recall using occurrence data presented in Table 2. The conditions by which food is prepared, as well as the brand, influence the content of AA, and it is thus difficult to assign a single AA concentration to all food of the same type. The mean concentration of the different pooled food items per category was used for the calculations in the present study.

**Figure 1:**
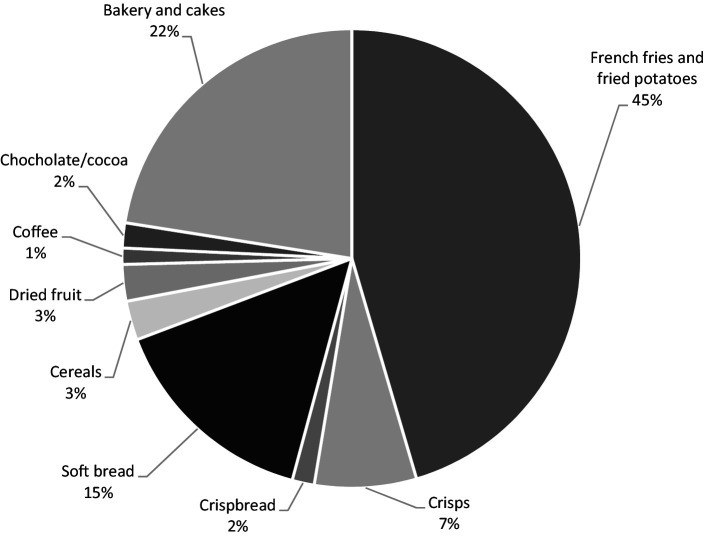
Contribution of different food groups to estimated mean daily acrylamide intake of Swedish adolescents participating in the study (school grades 5 and 8 and second grade of high school). Estimations are based on dietary recall data (2 days of registration) and occurrence data for acrylamide in the selected food groups.

The consumption pattern of AA-rich food, from the 2-day dietary recall, is similar across the age groups of adolescents. The majority of the adolescents stated that they consumed soft bread (94%), and about half consumed fried potatoes (49%), followed by bakery and cakes (41%) and cereals (35%). The intake pattern of AA is somewhat different from the food consumption pattern due to the different concentrations of AA in the different food categories. Considering all adolescents as a single group, fried potatoes/French fries contributed to the largest part of the AA intake (45%), followed by bakery and cakes (22%), bread (17%, soft bread/crispbread), and crisps (7%), as presented in Figure 1. A similar distribution was observed in all age groups separately, with the exception of dried fruit that was consumed by the youngest adolescents only (12 years old) (data not shown). From the 2-day dietary recall, the median intake was estimated to be 0.40 μg/kg bw/day (Table 4). The mean daily intake of AA was estimated to be 28 μg/day or 0.52 μg/kg bw (Table 4). The mean intake is in agreement with a previous estimation of the mean daily intake in Swedish adults (31 μg/day), calculated from occurrence data in foods at the Swedish market at that time (38). However, the major source of AA intake from food differs between the studies, where coffee is the largest exposure source among the Swedish adults. Due to the few coffee drinkers among the adolescents, the contribution is only 1%. The estimated daily intake is somewhat lower than the daily intake in adolescents calculated by EFSA (0.8 and 0.7 μg/kg bw in Sweden and Europe, respectively) (9). The estimated daily intake in Riksmaten adolescents is, however, closer to the mean intake in European adults (35 μg/day, corresponding to 0.5 μg/kg/day assuming 70 kg body weight) estimated by EFSA (9).

**Table 4:** Level of Hb adducts of acrylamide (AA), glycidamide (GA), and the sum of acrylamide and glycidamide (AA + GA) among eighth grade (15 years) and second grade (18 years) adolescents in relation to smoking habits.

		Daily smokers	Occasional smokers	Never smokers	Quitted smoking	Did not want to answer
Acrylamide Hb adducts (pmol/g Hb)	Mean ± *SD* (detection ratio)^a^	100 ± 40 (8/8)	39 ± 17 (47/50)	36 ± 17 (323/323)	34 ± 8 (9/9)	41 ± 22 (9/9)
	Median [min–max]	108 [42–156]	36 [17–100]	32 [14–225]	33 [20–45]	31 [24–87]
	P05–P95	42–146	20–65	20–62	23–43	25–79
Glycidamide Hb adducts (pmol/g Hb)	Mean ± *SD* (detection ratio)^a^	101 ± 74 (7/8)	53 ± 29 (36/50)	50 ± 35 (238/323)	35 ± 12 (6/9)	63 ± 45 (6/9)
	Median [min–max]	87 [26–255]	50 [10–125]	47 [9–453]	35 [20–51]	38 [30–126]
	P05–P95	34–213	19–110	22–83	21–50	30–124
AA + GA Hb adducts (pmol/g Hb)	Mean ± *SD* (detection ratio)^a^	200 ± 113 (7/8)	95 ± 44 (36/50)	88 ± 51 (238/323)	67 ± 11 (6/9)	109 ± 71 (6/9)
	Median [min–max]	208 [68–410]	94 [36–204]	80 [29–678]	68 [49–80]	72 [55–213]
	P05–P95	76–361	40–174	47–156	52–78	55–206

^a^Detection ratio refers to the number of samples (individuals) where the analyte was detected in relation to the total number of samples (individuals) per group (smoking status).

#### 3.2.2 Comparison of acrylamide intakes estimated from Hb adduct levels and from dietary recall

**Figure 2:**
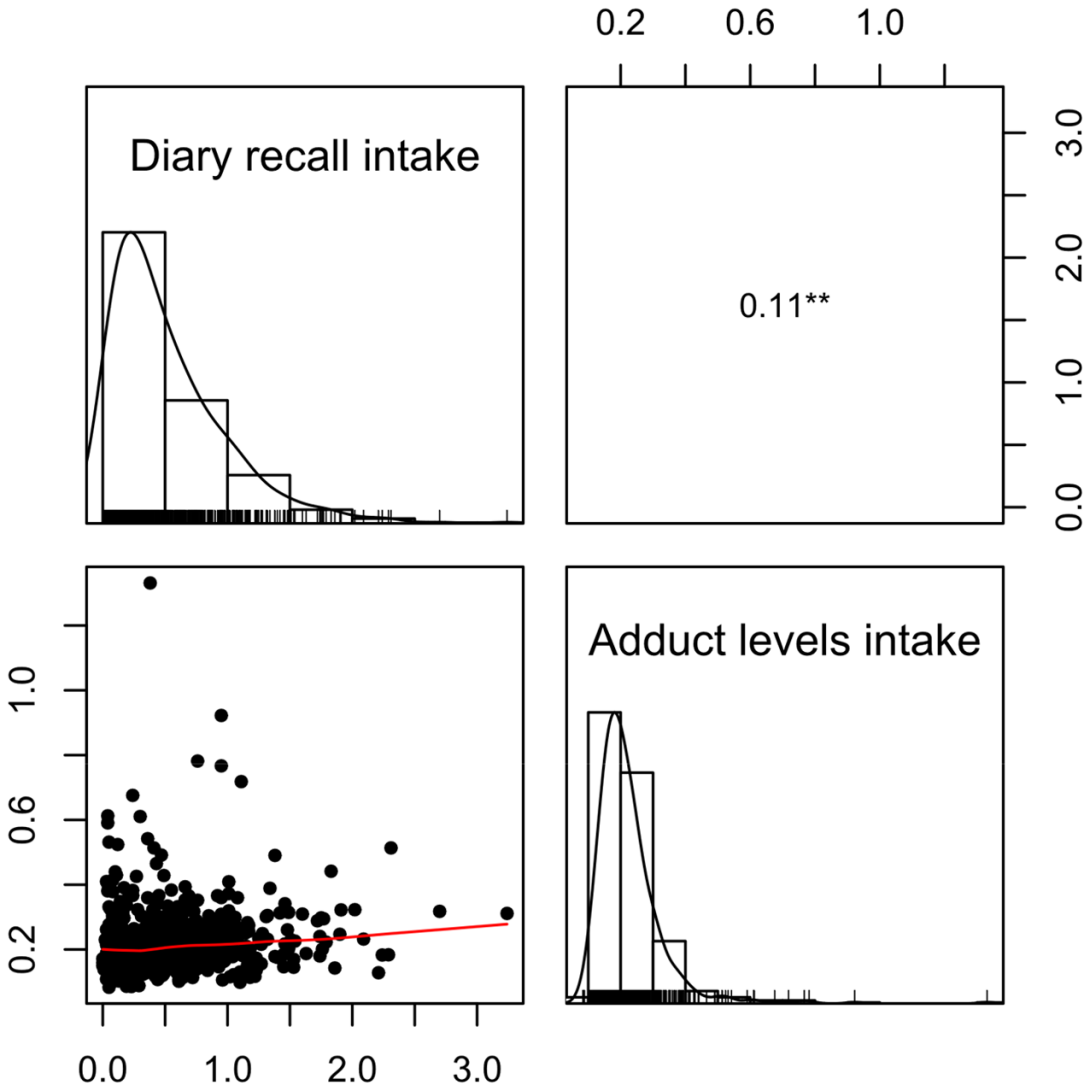
Spearman correlation plot for the comparison of the acrylamide (AA) intake estimates (μg AA/day and kg bw) for all adolescents from the 2-day dietary recall (diary recall intake) and AA Hb adduct levels (adduct levels intake) (*p* ≤ 0.05 with *, *p* ≤ 0.01 with **, and *p* ≤ 0.001 with ***).

The intake of AA per kg bw was also estimated by converting the AA adduct levels in Table 3 to internal dose (AUC) and using data from an earlier dietary intervention study (33). The mean AA intake estimated from AA adduct levels, for the whole group of adolescents, was thereby estimated to be 0.23 μg AA/kg bw/day (Table 4), which is about half of the intake estimated from the dietary recall. In comparison of the intake estimates from AA adduct levels and from the dietary recall (Figure 2), a significant weak positive correlation was observed (Spearman rank correlation; *p*-value = 8 × 10^−3^; *ρ* = 0.11).

It was also studied whether the estimated AA intake from selected food groups was associated with the Hb adduct levels of AA, GA, and AA + GA. Significant Spearman correlation was detected (*p*-value = 0.015; *ρ* = 0.10) only for the food category fried potatoes/French fries.

Hb adduct levels reflect the exposure during the last four months (the lifetime of erythrocytes). The calculated total daily AA intakes, based on the dietary recall, reflect the intake from two non-consecutive days in one week. Food frequency questions reflect general food consumption over a longer time period. Therefore, it might be expected that intake data from food frequency questionnaires would have a better correlation to Hb adduct levels than intake data from dietary recalls. In support of this, Brisson et al. (39) earlier observed that Hb adduct levels from AA were more strongly associated with intake estimates obtained from food frequency questionnaires reflecting dietary intake over a longer time period than from intake estimates from a food diary over a few days.

#### 3.2.3 Consumption frequency of acrylamide-rich food and associations with Hb adduct levels

**Figure 3:**
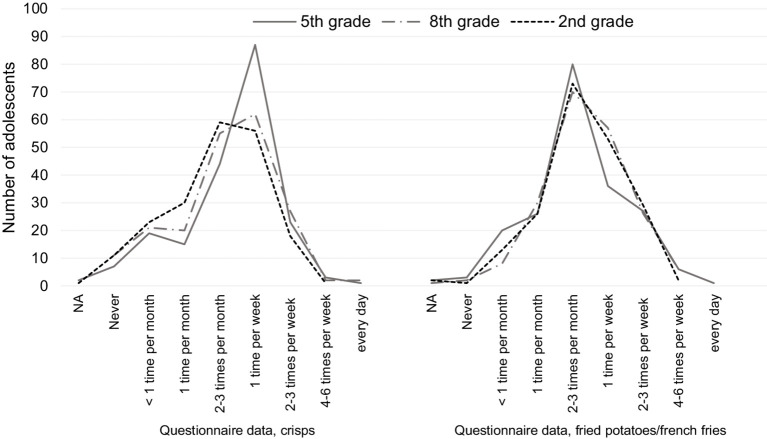
Number of adolescents with different consumption frequencies of crisps and fried potatoes/French fries, extracted from questionnaire data for adolescents in fifth grade (12 years), eighth grade (15 years), and second grade of high school (18 years).

The Hb adduct levels of AA and of the sum AA + GA were compared to consumption frequencies (over the last 12 months) of fried potatoes/French fries, bread, and crisps obtained from the web questionnaire (Figure 3; data for bread not shown). The adolescents were divided into groups based on consumption frequency. For crisps, a significant difference could be observed only for AA Hb adduct levels among the 18-year-old adolescents (second grade of high school), using the Kruskal–Wallis *H* test (*p*-value = 0.03). Further investigation with Dunn’s test revealed statistical significance between the group that answered “less than 1 time per month or 1 time per month” and both groups that answered that they had higher consumption (“2–3 times per month to 1 time per week,” and “2–3 times per week to 6 times per week or every day”), with higher AA Hb adduct levels in the groups with higher consumption. However, applying the Bonferroni correction produced non-significant results (Bonferroni corrected *p*-values = 0.07 and 0.06, respectively). The boxplots of consumption frequencies of crisps are included in Supplementary Figure S3. For fried potatoes/French fries, a significant difference in AA adduct levels was found for the 15-year-old adolescents (eighth grade) using Kruskal–Wallis (*p*-value = 1.4 × 10^−4^). Further investigation with Dunn’s test showed a significant difference in pairwise comparison between “less than 1 time per month or 1 time per month,” and those with higher consumption (“2–3 times per month to 1 time per week,” and “2–3 times per week to 6 times per week or every day”) (Supplementary Figure S4).

As reviewed by Pedersen et al. (13), crisps have been found to be the most common foodstuff to correlate with AA intake/AA adducts, as well as coffee or crisp bread (40–42).

#### 3.2.4 The basis for estimation of acrylamide intake from Hb adduct levels and dietary consumption data

For the calculation of AA intake (Table 4) from Hb adduct levels, data were used from a previous dietary intervention study with adults consuming AA-rich food for 4 days (32, 33). Although that study was performed with few participants, the results showed a clear daily increase in adduct levels and a related calculated internal dose (AUC). It would be valuable to conduct a similar intervention study in adolescents to confirm whether the same internal dose of AA per exposure dose of AA is observed as for the adults in the previous study. Compared to corresponding estimates extrapolated from rats or humans exposed to high AA doses via drinking water (43, 44), the estimate from the previous human dietary intervention study is an improvement. We assumed the same internal dose of AA per exposure dose of AA for adolescents as earlier obtained for adults, and using that data, we arrived at half of the median AA intake from the measured AA adduct levels compared to the estimate from the dietary recall data (0.20 vs. 0.40 μg/kg bw/day).

The dietary survey Riksmaten Adolescents 2016–17 was not designed to specifically investigate AA exposure, but still significant correlations could be observed between estimated AA intakes and Hb adduct levels. For a better quantitative description of the relation between AA intake from different foods and AA Hb adduct levels, a study with another design would be needed. The optimal study design would be a duplicate diet study over a short period, where AA is measured in duplicate portions of all consumed food and drinks by the studied individuals, in combination with blood sampling before and after, followed by Hb adduct analysis, similar to the intervention study mentioned above. However, if not performed as an intervention study for short time and with few individuals, duplicate diet studies are very expensive and not practical for studying the dietary habits during longer time periods. More feasible types of studies would be to use dietary questionnaires or dietary registrations with a specific focus on the foods known to contain AA.

In a recent study of a smaller group of adults (*n* = 142) by Vryonidis et al. (34), the AA intakes estimated from Hb adduct levels were compared with AA intakes estimated by probabilistic modelling from food frequency questionnaire and a 2-day food diary. The comparison at an individual level showed no large bias^1^ between the three methods, and the mean/median intakes obtained with food frequency questionnaire and food diary, as well as from Hb adducts, were about the same. This gives support that probabilistic modelling for estimation of intake from food consumption data would give a better estimate of the intake than deterministic calculation at the group level. In addition, it was discussed that the results obtained in that study, showing similar AA intake estimates from food consumption data and from AA Hb adduct levels, did not give support for a large contribution to internal dose from endogenous production of AA, as earlier suggested (0.3–0.4 μg/kg bw/day) (14). The present study could not exclude endogenous production of AA but does not support a large contribution from any endogenous production to the internal dose of AA, as the internal dose from total exposure to AA as estimated from the AA adduct level is lower than expected from estimated dietary exposure.

### 3.3 Hb adduct levels in relation to sociodemographic factors and smoking habits

#### 3.3.1 Sex, age groups, type of residence, and parental education

No statistically significant difference was observed in Hb adduct levels (AA, GA, or AA + GA) between female and male participants for any school grade (see also Supplementary Figure S5). Neither the type of residence (urban or rural) nor the educational level of the parents showed any significant differences with the levels of Hb adducts (data not shown).

**Figure 4:**
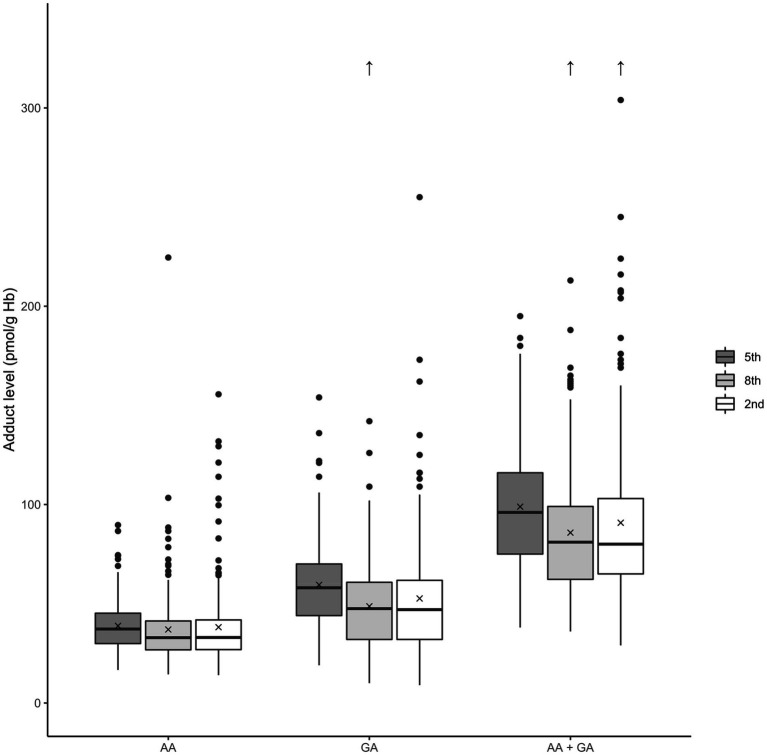
Box plots of the Hb adduct levels among adolescents in different school grades. The level of Hb adducts of acrylamide (AA), glycidamide (GA), and the sum of the adducts (AA + GA) is described; school grades: fifth grade (12 years), eighth grade (15 years), and second grade of high school (18 years). The box denotes the interquartile range (IQR), with the lower quartile (Q1, 25th percentile), the median, which is the second quartile (Q2, 50th percentile), and the upper quartile (Q3, 75th percentile). The whiskers show a range of ±1.5 times the IQR. The values beyond the range of the whiskers are outliers and are marked with ●. The mean is denoted by X, and outlier values over 320 pmol/g Hb are shown as ↑.

The younger adolescents (fifth grade, 12 years) did appear to have somewhat higher, although not statistically significant, AA, GA, and AA + GA adduct levels than the older adolescents (15 and 18 years old; Figure 4). With regard to published data, AA Hb adduct levels in adolescents from different countries show no clear difference in comparison to data from adults (reviewed by (13)).

#### 3.3.2 Cigarette smoking

Cigarette smoke is a known exposure source for AA (12), which has been measured as Hb adducts (*cf.* (45)), and therefore the exposure levels were compared between smokers and non-smokers. The youngest participants (12 years old) did not get any questions in the survey about their smoking habits. The great majority of the older adolescents (81%) who were asked questions regarding smoking (the higher grades, 15 and 18 years) stated that they had never smoked. Approximately 13% of these adolescents stated that they were occasional smokers, approximately 2% stated that they quit smoking, and 2% were daily smokers (approximately 2% of the adolescents asked, did not want to answer) (see Table 1).

**Figure 5:**
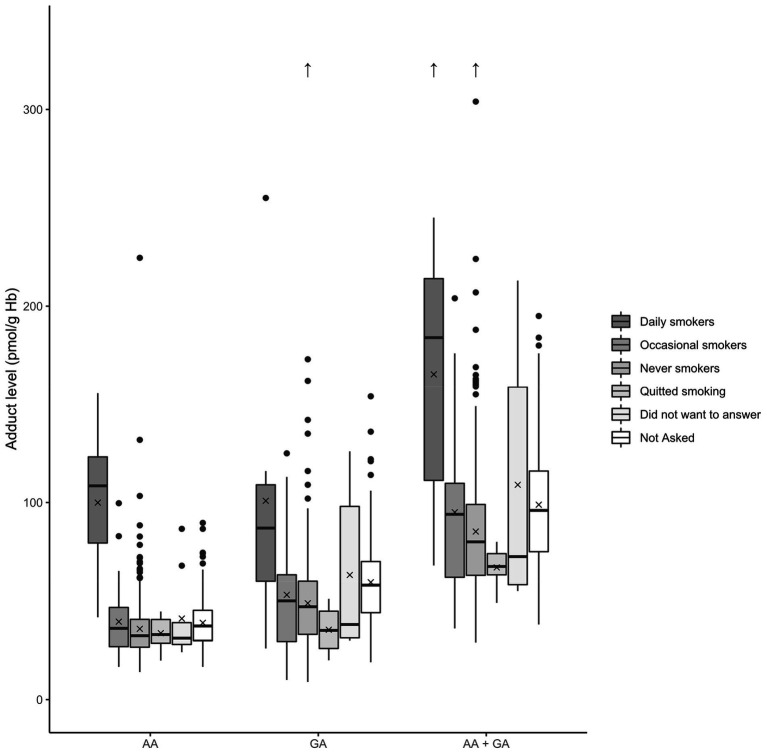
Box plots of the blood Hb adduct levels in relation to the smoking status of the adolescents. The levels of Hb adducts of acrylamide (AA), glycidamide (GA), and the sum of the adducts (AA + GA) are described. Note that “Not Asked” refers to fifth grade (12 years old) students where the question was not included in the survey. The box denotes the interquartile range (IQR), with the lower quartile (Q1, 25th percentile), the median, which is the second quartile (Q2, 50th percentile), and the upper quartile (Q3, 75th percentile). The whiskers show a range of ±1.5 times the IQR. The values beyond the range of the whiskers are outliers and are marked with ●. The mean is denoted by X, and outlier values over 320 pmol/g Hb are shown as ↑.

The results with respect to smoking habits of the older adolescents (15 and 18 years old) are presented in Table 5 and Figure 5. Statistical analysis to investigate differences between the smoking groups (excluding fifth grade, 12 years) was initially done by Kruskal–Wallis one-way analysis of variance. Median AA adduct levels in the groups of daily smokers, occasional smokers, and never smokers were 108, 36, and 32 pmol/g Hb, respectively. A clear increase can be observed for the adduct levels of the daily smokers in Figure 5. Using the Mann–Whitney *U* test revealed significant differences between daily smokers and never smokers for AA, GA, and AA + GA adduct levels (*p*-values = 2 × 10^−5^, 0.01, and 2 × 10^−3^, respectively), with higher levels in daily smokers than compared to in never smokers. In contrast, no such differences were observed for occasional smokers, which was confirmed with Mann–Whitney *U* tests (data not shown). In published studies, higher levels of AA Hb adducts are generally monitored in smokers, but with large variations between the smoking individuals. An increase of up to approximately 6 pmol/g Hb per smoked cigarette a day has been found in earlier studies [reviewed by (13)]. In the present study, no information was available about the number of smoked cigarettes among the daily and occasional smokers.

**Table 5:** Estimated daily intake of acrylamide (AA) in (μg/day) and within parenthesis in (μg/kg body weight per day) among adolescents in fifth grade (12 years), eighth grade (15 years), and second grade of high school (18 years).

		AA intake estimated from dietary recall (μg AA per day)			AA intake estimated from dietary recall (μg AA per day and kg bw)			AA intake estimated from Hb adducts (μg AA per day and kg bw)		
		Mean ± *SD* (data availability ratio)	Median[min–max]	P05–P95	Mean ± *SD* (data availability ratio)	Median[min–max]	P05–P95	Mean ± *SD* (detection ratio)	Median[min–max]	P05–P95
All grades (n = 600)	All	28 ± 22 (600/600)	22[0–128]	3–71	0.52 ± 0.46 (600/600)	0.40 [0–3.24]	0.05–1.45	0.23 ± 0.11 (594/600)	0.20 [0.08–1.33]	0.12–0.38
	♀	24 ± 18 (300/300)	20[0–105]	3–58	0.48 ± 0.39 (300/300)	0.36 [0–1.91]	0.05–1.31	0.22 ± 0.1 (298/300)	0.20 [0.09–0.92]	0.12–0.38
	♂	31 ± 26 (300/300)	24[0–128]	3–79	0.57 ± 0.52 (300/300)	0.43 [0–3.24]	0.05–1.60	0.23 ± 0.11 (296/300)	0.20 [0.08–1.33]	0.12–0.40
Fifth grade (n = 201)	All	28 ± 23 (201/201)	23[1–125]	3–67	0.69 ± 0.58 (201/201)	0.55 [0.02–3.24]	0.07–1.76	0.23 ± 0.08 (198/201)	0.22 [0.10–0.53]	0.13–0.37
	♀	25 ± 18 (100/100)	21[1–80]	3–58	0.62 ± 0.48 (100/100)	0.44 [0.02–1.91]	0.07–1.49	0.23 ± 0.07 (98/100)	0.22 [0.10–0.44]	0.14–0.37
	♂	31 ± 26 (101/101)	25[1–125]	4–84	0.75 ± 0.66 (101/101)	0.59 [0.02–3.24]	0.06–2.02	0.23 ± 0.08 (100/101)	0.22 [0.11–0.53]	0.13–0.35
Eighth grade (n = 200)	All	26 ± 20 (200/200)	21[0–119]	2–63	0.45 ± 0.36 (200/200)	0.38 [0–2.21]	0.04–1.23	0.22 ± 0.11 (200/200)	0.20 [0.09–1.33]	0.12–0.38
	♀	21 ± 15 (100/100)	16[0–78]	2–46	0.36 ± 0.27 (100/100)	0.30 [0–1.45]	0.04–0.78	0.21 ± 0.08 (100/100)	0.19 [0.09–0.61]	0.12–0.37
	♂	32 ± 24 (100/100)	28[0–119]	3–74	0.54 ± 0.42 (100/100)	0.45 [0–2.21]	0.05–1.32	0.23 ± 0.14 (100/100)	0.20 [0.09–1.33]	0.12–0.43
Second grade (n = 199)	All	29 ± 24 (199/199)	22[0–128]	3–76	0.43 ± 0.37 (199/199)	0.32 [0–2.09]	0.04–1.08	0.23 ± 0.12 (196/199)	0.20 [0.08–0.92]	0.12–0.41
	♀	27 ± 20 (100/100)	25[1–105]	3–60	0.45 ± 0.34 (100/100)	0.35 [0.02–1.74]	0.04–1.08	0.24 ± 0.13 (100/100)	0.20 [0.10–0.92]	0.12–0.40
	♂	31 ± 27 (99/99)	22[0–128]	4–79	0.42 ± 0.39 (99/99)	0.28 [0–2.09]	0.05–1.06	0.22 ± 0.12 (96/99)	0.19 [0.08–0.77]	0.11–0.40
Previous Studies	(38)	31	[0–132]	9.1–62						
	(9)					0.7 [0.4–0.9]^a^	1.4 [0.9–2.0]^b^			

The dietary recall intakes are based on the mean consumption of selected food groups (bakery and cakes, soft bread, cereals, chocolate/cocoa products, coffee, crispbread, crisps, dried fruit, French fries, and fried potatoes) during the 2 days of dietary recall, occurrence data in these food groups, and the body weight of the participants. The intakes from Hb adducts are calculated from the relationship between known AA intake and internal dose (from a previous study; see Section 2.6). ^a^Median [min–max] for adolescents of the mean across different surveys (*n* = 16). ^b^Median [min–max] for adolescents of the P95 across different surveys (*n* = 16).

### 3.4 Methodological differences of the approaches for estimation of acrylamide intake

Riksmaten adolescents 2016–17 is a large, nationwide study providing nationally representative data on exposure to AA in Swedish adolescents. The study is unique in that it combines a detailed dietary survey with blood sampling, thus making it possible to investigate associations between consumption of AA-rich foods or calculated intakes of AA and blood concentrations. However, the survey was not specifically designed to study AA exposure, and the three approaches utilized in the investigation of AA exposure via food do have differences and all have limitations regarding the exposure estimation.

Regarding Hb adducts as biomarkers of exposure, it is important to consider that it is the exposure during the lifetime of the erythrocytes (about 126 days) that is reflected by the measured adduct levels. Due to the disappearance of old erythrocytes, exposure during the latest time period before sampling has higher impact on the adduct level (see, e.g., (46)). At about constant exposure, the adduct level could be used to estimate the mean internal dose (AUC) or mean exposure during the last four months. Regarding adduct levels from AA and GA from dietary exposure, the intra-individual variation over time, including different seasons, is low compared to the inter-individual variation (16). One limitation for many studied adducts is that they could originate from several sources, which contribute to the measured adduct levels. For AA, the Hb adducts measured are the reaction product with the N-terminal Val of AA from all possible sources and not just from food. Tobacco could also contribute to AA exposure and the adduct level (45), and distinguishing between the exposure sources of AA is not feasible. In the literature, it is also supported that endogenous production of AA could contribute to internal dose/exposure (14), which would also contribute to the adduct level measured.

In contrast to the adducts, the estimation of AA exposure from just food is more straightforward, i.e., using dietary recall data and corresponding AA concentrations in consumed food items. However, data that rely on individuals answering questions about their diet also have drawbacks. Here, data from the 2-day dietary recall only reflect 2 days of the individuals’ diet, which may not be representative of their diet and intake of AA in general. The web-based questionnaires, also used in this study, could be expected to better reflect general consumption over a long period of time. In addition, the food frequency questions used in the present study were designed to reflect seasonal variation in consumption rather than intake of AA, which is reflected by adduct levels in the blood.

Moreover, the intakes estimated from dietary recall data in combination with concentrations in food rely on laboratory analysis of food where the AA content is measured. The AA content in food can vary greatly, and analysis is usually limited to commercially available food that ignores home cooking. In this study, we used the mean of measured AA content in food (using values from food items bought in Sweden, supplemented with values from the scientific opinion by EFSA; see Section 2.4), which would be accurate only if the participants consumed the average food.

### 3.5 Cancer risk estimates

The potential cancer risk is an issue with regard to exposure to AA from food and has been a theme for many scientific publications. Therefore, the question of what the median AA intake obtained in the present study could mean with regard to cancer risk, using available approaches and unit cancer risk estimates for AA, is of interest to discuss. Bioassays showed in the 1980s that AA is carcinogenic in animals (47, 48). Complete chronic cancer bioassays with rats and mice exposed via drinking water to AA (49) or to GA (50), later performed by the US National Toxicology Program (US NTP), have led to the conclusion that the carcinogenic activity of AA is due to the genotoxic metabolite GA (9, 50).

The margin of exposure (MOE) approach was used to characterize the cancer risk for the adolescents in the present study. The MOE values were calculated from the ratio of the observed AA intake obtained in the present study to the benchmark dose lower confidence limit of 10%, BMDL₁₀ (point of departure), of 0.17 mg/kg body weight (bw) per day for neoplastic effects in male mice, obtained from the cancer bioassays performed by US NTP (9, 51). Based on the median estimated AA intake (μg/kg bw/day) for all participants from the dietary recall, the MOE was calculated to be 430, and the corresponding AA intake estimated from the Hb adducts resulted in a MOE value of 835. For substances that are carcinogenic and genotoxic, a MOE value of 10,000 or higher is considered to be of low concern for public health, according to the EFSA. The calculated MOE values from the medians of any grouping of the data (grade/sex/parental education/type of residency) in the present study are significantly lower than 10,000 and would therefore indicate a concern. This result is in accordance with earlier data from the European population, even if MOE is somewhat higher in the present study due to a lower AA intake than earlier estimated for adolescents (9).

There are also quantitative estimates of cancer risk from AA obtained by extrapolation from the rodent carcinogenicity studies. The published cancer risk estimates for AA are based on the results of the early cancer bioassays in rats mentioned above (52–55), except for the estimate by (56), which used the later US NTP studies of carcinogenicity. The estimates for cancer risk per exposure dose of AA differ by more than a factor of 10 since different models are used for the calculations. US EPA (54) lowered their previous unit cancer risk estimate, and of the models mentioned above, their model gives the lowest unit cancer risk estimate of AA. The US EPA estimate is a slope factor for oral exposure of 0.5 per mg/kg bw/day, which can be expressed as a lifetime risk of 0.5 cases per 1,000 persons at an exposure to 1 μg of AA/kg bw/day during lifetime (estimated from two types of tumors in male rats) (54). This would correspond to 0.1–0.2 cases/1000 persons at life-long exposure at the median intakes of 0.2 or 0.4 μg AA/kg bw observed with the different methods in the present study.

The highest estimate is based on a multiplicative (relative) risk model (53). This model is similar to the one used for ionizing radiation, which uses background tumor frequency and the internal dose (AUC) of the genotoxic agent for risk extrapolation (57). The derived risk coefficient is expressed as the internal dose that doubles the background tumor frequency and has been shown to be almost independent of tumor site, sex, and species for the chemicals tested with this model (53, 57–59). The doubling dose (for multiple sites) for AA exposure, estimated from the early cancer bioassays in rats, is *ca.*13 mM × h of GA (the genotoxic metabolite), which corresponds to an intake of approximately 500 mg/kg bw of AA for rats (53) (re-evaluation ongoing). For humans, the same doubling dose of GA would correspond to about half of that AA intake (33). The observed median intakes in the present study, 0.2 or 0.4 μg AA/kg bw/day during a lifetime (70 years), would correspond to a total intake of approximately 5 or 10 mg AA/kg bw. This would correspond to a few percent of the doubling dose of AA in humans, thus representing an estimated relative risk.

Despite the large quantitative differences, the risk estimates obtained by different models all indicate that increased cancer risks from life-long exposures to AA from food are of concern. A reliable cancer risk estimate from toxicological studies would be of great value for assessing how important it is to reduce AA intake in the general population. This should include improvements in the risk models used (60). It has been discussed earlier that with unit cancer risk estimates for AA, similar to the above, the estimated extra lifetime cancer risk for a population with an increased lifetime exposure of 0.4 μg AA/kg bw/day will be low (up to a few percent relative risk) compared to the risk usually detectable in epidemiological studies (61, 62).

With regard to results from epidemiological studies, the association between dietary AA intake and cancer is not clear [see, e.g., review by (63)]. The most recent meta-analysis (64) showed no associations of dietary AA exposure and site-specific non-gynecological cancers. One meta-analysis of breast and gynecological cancers in females found positive associations with AA intake (65), and another meta-analysis of women’s cancer did not find any significant association (66). A later study found a positive association between breast cancer in females and AA intake (67). These contradictory results have recently been discussed (68).

## 4 Conclusion

This study shows that exposure to acrylamide (AA) is widespread among Swedish adolescents. The distribution of AA intake is much wider, as estimated from the dietary recall data, than estimated from the adduct levels. Fried potatoes/French fries, crisps, and bread are known major sources of exposure to AA, and the consumption of these food categories was common among the studied adolescents. Although the dietary recall was not designed to study AA exposure specifically, a significant positive correlation was observed between estimated total dietary AA intake and hemoglobin adduct levels in the blood and the intake of certain AA-rich foods and hemoglobin adduct levels. In addition, smokers had significantly higher adduct levels in their blood than non-smokers. The intake of AA estimated from the dietary recalls was approximately twice as high as the intake estimated from the hemoglobin adduct levels in the blood. This discrepancy should be addressed in a future study aiming at studying AA exposure in children/adolescents, such as, e.g., a duplicate diet study in combination with adduct measurement and laboratory analysis of the consumed food. This is thought to be helpful to investigate true correlations and to get a better estimation of the contribution of food to the overall cancer risk.

The estimated AA intake from food consumption, together with the corresponding cancer risk estimates obtained from toxicological approaches, indicate that it is important to further decrease the exposure to AA in food. The large variation between individuals, as observed both from food consumption data and from measured AA adduct levels, emphasizes that it would be important that high consumers reduce their AA intake.

## Data availability statement

The datasets presented in this article are not readily available because the datasets contain personal data Requests to access the datasets should be directed to jenny.aasa@slv.se.

## Ethics statement

The studies involving humans were approved by Regional Ethical Review Board in Uppsala (No. 2015/190). The studies were conducted in accordance with the local legislation and institutional requirements. Written informed consent for participation in this study was provided by the participants’ legal guardians/next of kin.

## Author contributions

EV: Data curation, Formal Analysis, Investigation, Methodology, Software, Validation, Visualization, Writing – original draft, Writing – review & editing. MT: Conceptualization, Funding acquisition, Investigation, Project administration, Resources, Supervision, Writing – original draft, Writing – review & editing. SL: Data curation, Writing – review & editing, Funding acquisition, Investigation, Methodology, Project administration. JR: Data curation, Writing – review & editing, Investigation, Methodology. JA: Conceptualization, Data curation, Formal analysis, Funding acquisition, Investigation, Project administration, Resources, Supervision, Visualization, Writing – original draft, Writing – review & editing.

## Footnotes

^1^Bias in this case denotes the average difference from the participants between the intake estimates of two methods.
