# Supplementary material for: Estimation of intake and quantification of hemoglobin adducts of acrylamide in adolescents in Sweden
Source: Front Nutr. 2024 Jun 3;11:1371612. doi: 10.3389/fnut.2024.1371612 (PMC11180753; doi:10.3389/fnut.2024.1371612)

Supplementary Material

Estimation of intake and quantification of haemoglobin adducts of acrylamide in blood samples from adolescents in Sweden

Efstathios Vryonidis[1]*, Margareta Törnqvist[1], Sanna Lignell[2], Johan Rosén[3], Jenny Aasa[2]*

[1]Department of Environmental Science, Stockholm University, Stockholm, Sweden

[2] Division of Risk and Benefit Assessment, Swedish Food Agency, Uppsala, Sweden

[3] Division of Laboratory Investigation and Analysis, Swedish Food Agency, Uppsala, Sweden

*** Correspondence:**
Corresponding Author
jenny.aasa@slv.se, efstathios.vryonidis@aces.su.se

1 Supplementary Figures and Tables

1.1 Complimentary instrumental information

1.1.1 LC program

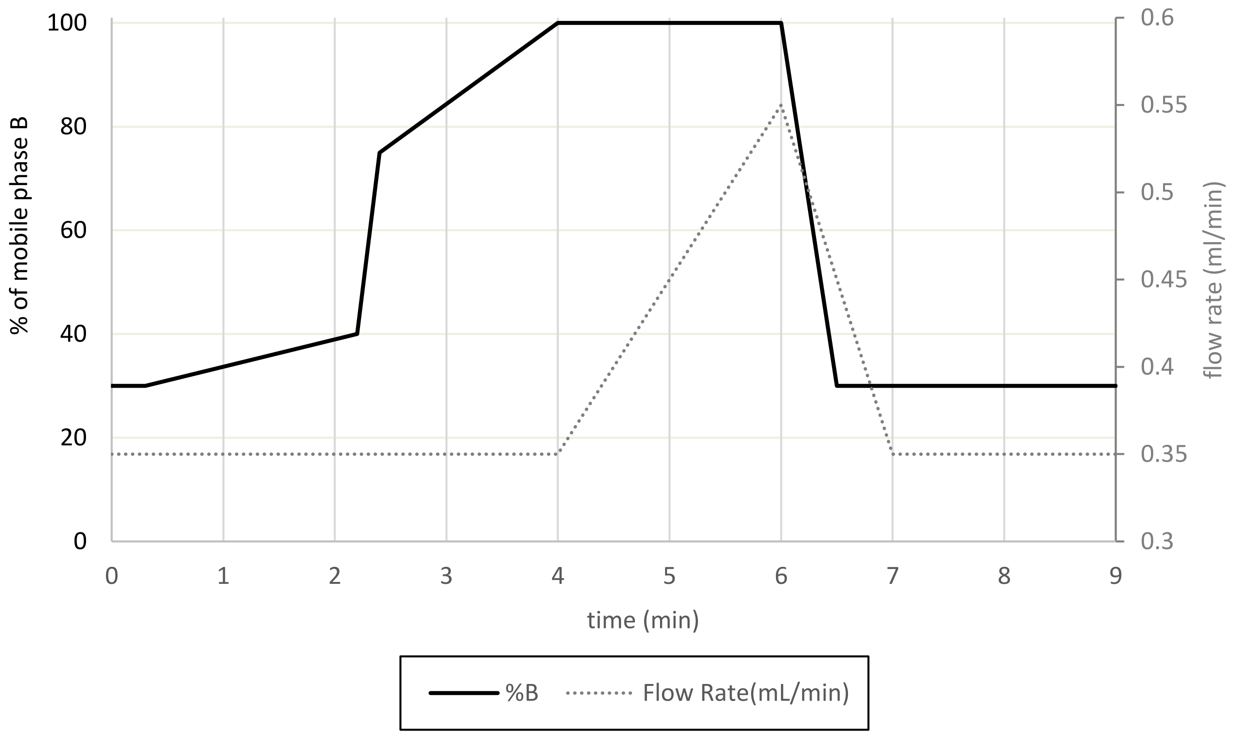

Supplementary Figure 1. Gradient program used for this study. Note that at t=0 min, when the injection is made, the flow is diverted to waste, the flow is switched to the MS during t=1.99 min to t=3.9 min, thereafter the flow is switched back to waste. From t=4 min to t=6 min the program flushes the chromatographic column with mobile phase B and while increasing the flow (up to 0.55 ml/min). from t= 6 min to t=9 min, the flow and composition of the mobile phase gradually returns to the initial conditions of the program to equilibrate the column and stabilize the column pressure before a following injection.

1.1.2 MS transitions and monitored analytes

Supplementary Table 1. List of the fragments monitored by multiple reaction monitoring for this study. AA refers to the part of the analyte that derives from acrylamide, GA refers to the part of the analyte that derives from glycidamide, Val refers to the part of the analyte that comes from the amino acid valine, FTH refers to the class of the analytes which is fluorescein thiohydantoins and it is the part of the analyte that comes from fluorescein isothiocyanate (the derivatization regent). See also Supplementary Figure 2 below, for the related structures of the precursor $[M+H]^+$ ions.

**Transition**	**Cone Volt.**	**Col. Energy**	**Compound**
560 ⟶ 445	80	40	AA-Val-FTH
560 ⟶ 489	80	40	AA-Val-FTH
560 ⟶ 517	80	30	AA-Val-FTH
563 ⟶ 445	80	40	AA(d_3)-Val-FTH
563 ⟶ 489	80	40	AA(d_3)-Val-FTH
563 ⟶ 520	80	30	AA(d_3)-Val-FTH
567 ⟶ 445	80	40	AA-Val(d_7)-FTH
567 ⟶ 496	80	40	AA-Val(d_7)-FTH
567 ⟶ 517	80	30	AA-Val(d_7)-FTH
576 ⟶ 489	60	40	GA-Val-FTH
576 ⟶ 531	60	40	GA-Val-FTH
576 ⟶ 533	60	40	GA-Val-FTH
583 ⟶ 489	60	40	GA-Val(d_7)-FTH
583 ⟶ 533	60	40	GA-Val(d_7)-FTH
583 ⟶ 538	60	40	GA-Val(d_7)-FTH

1.1.3 Precursor $[M+H]^+$ ions of the analytes

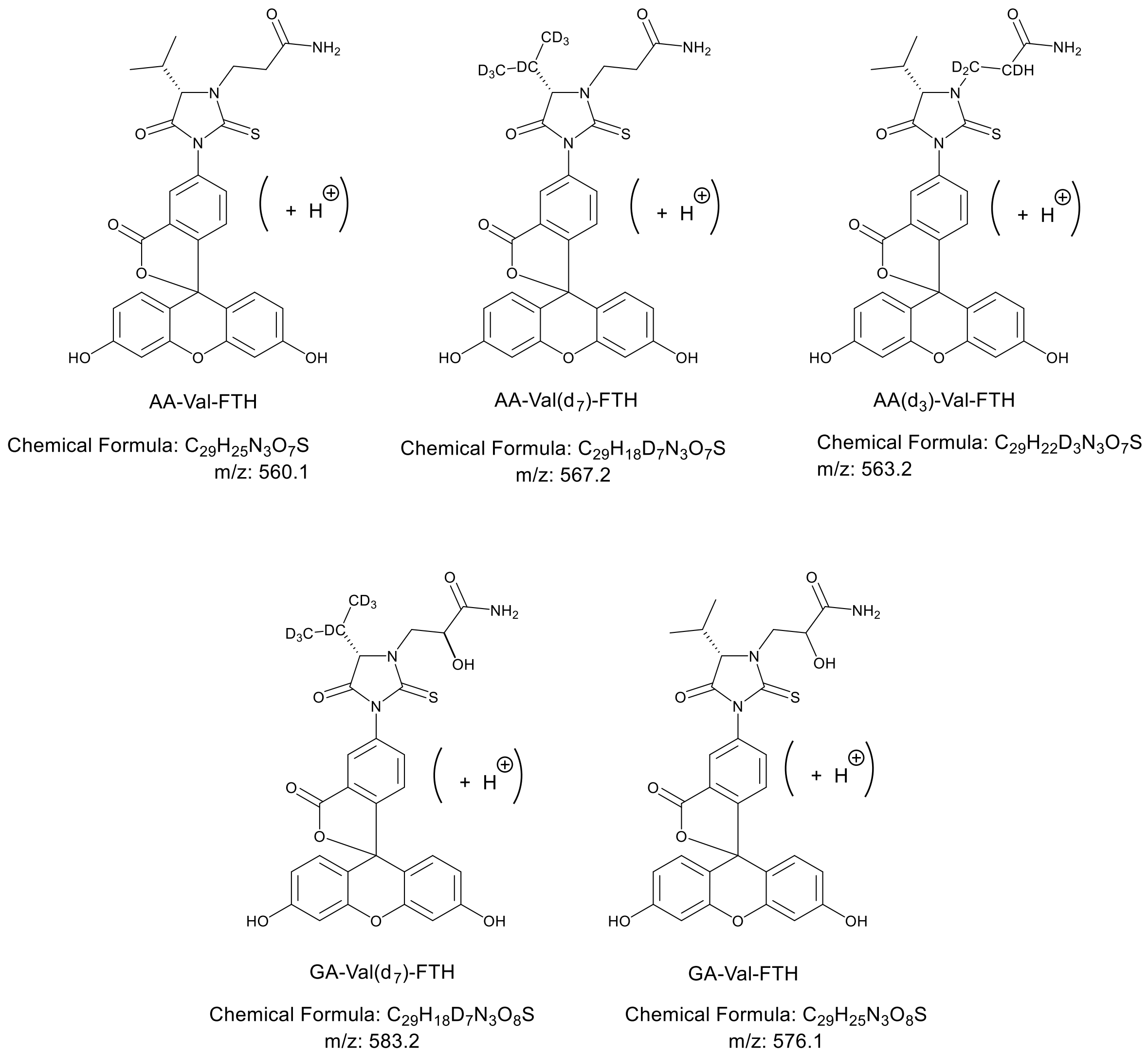

Supplementary Figure 2. Structure of the main molecular ion for each analyte monitored in MS. AA refers to the part of the analyte that derives from acrylamide, GA refers to the part of the analyte that derives from glycidamide, Val refers to the part of the analyte that comes from the amino acid valine, FTH refers to the class of the analytes which is fluorescein thiohydantoins and it is the part of the analyte that comes from fluorescein isothiocyanate (the derivatization regent). Note that fluorescein is drawn as its lactone tautomer. Only the major reaction product is drawn for the analytes that come from GA/GA(d_7), however two products are expected (and observed) as the asymmetrical epoxide GA/GA(d_7) reacts by SN2 with Val.

2 Complimentary data processing information

2.1 Calculation of batch correction factor

The formula of the batch correction factor used for normalizing the day-day variability

$$batch\ correction\ factor\ for\ batch\ x = \frac{\left(\frac{mean\ x_{QC\ low}}{mean\ CC_{QC\ low}} + \frac{mean\ x_{QC\ medium}}{mean\ CC_{QC\ medium}} + \frac{mean\ x_{QC\ high}}{mean\ CC_{QC\ high}}\right)}{number\ of QClevels (= 3)}$$

3 Complimentary results

3.1 Two-day dietary recalls

Supplementary Figure 3*. Box and whiskers plot of the Hb adduct levels among female and male adolescents in different school grades.* *The level of Hb adducts of acrylamide (AA), glycidamide (GA) and the sum of the adducts (AA+GA) is described; school grades; 5th grade (12 years), 8th grade (15 years) and 2nd grade of high school (18 years). The box denotes the interquartile range (IQR), with the lower quartile (Q1, 25th percentile), the median which is the second quartile (Q2, 50th percentile) and the upper quartile (Q3, 75th percentile). The whiskers show the range of ± 1.5 times the IQR. The values beyond the range of the whiskers are outliers and are marked with •. The mean is denoted by ×, and outlier values over 320 pmol/g Hb are shown as ↑.*

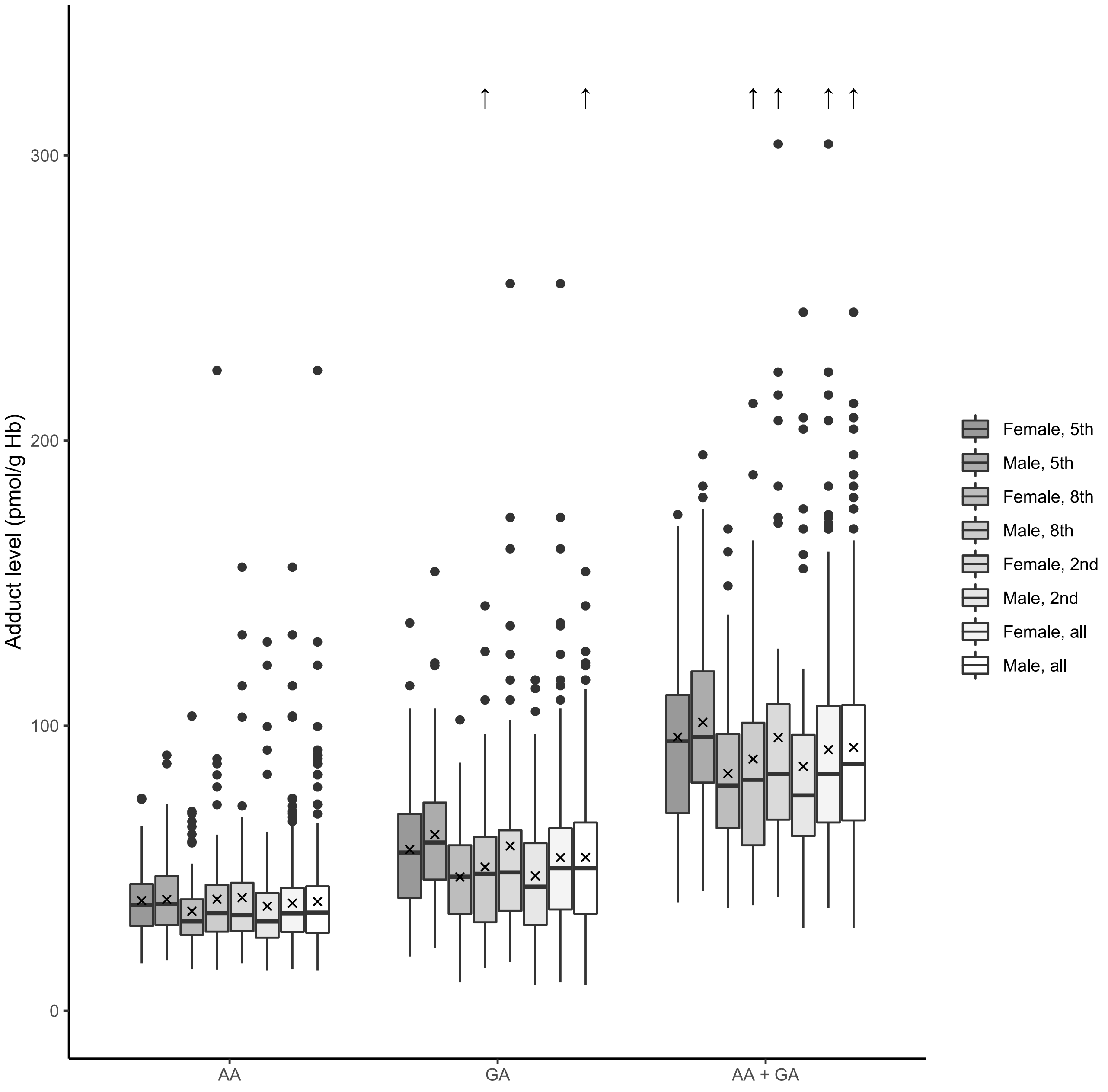

3.2 Web-based frequency questionnaires

Supplementary Figure 4. ***Box plots of the Hb adduct levels in adolescents with different consumption frequencies of potato crisp.*** *The level of Hb adducts of acrylamide (AA), glycidamide (GA), and the sum of acrylamide and glycidamide (AA+GA) is shown in relation to different crisps consumption frequencies for students at 5th grade (12 years), 8th grade (15 years) and 2nd grade of high school (18 years). The box denotes the interquartile range (IQR), with the lower quartile (Q1, 25th percentile), the median which is the second quartile (Q2, 50th percentile) and the upper quartile (Q3, 75th percentile). The whiskers show the range of ± 1.5 times the IQR. The values beyond the range of the whiskers are outliers and are marked with ●. The mean is denoted by ×, and outlier values over 320 pmol/g Hb are shown as ↑.*

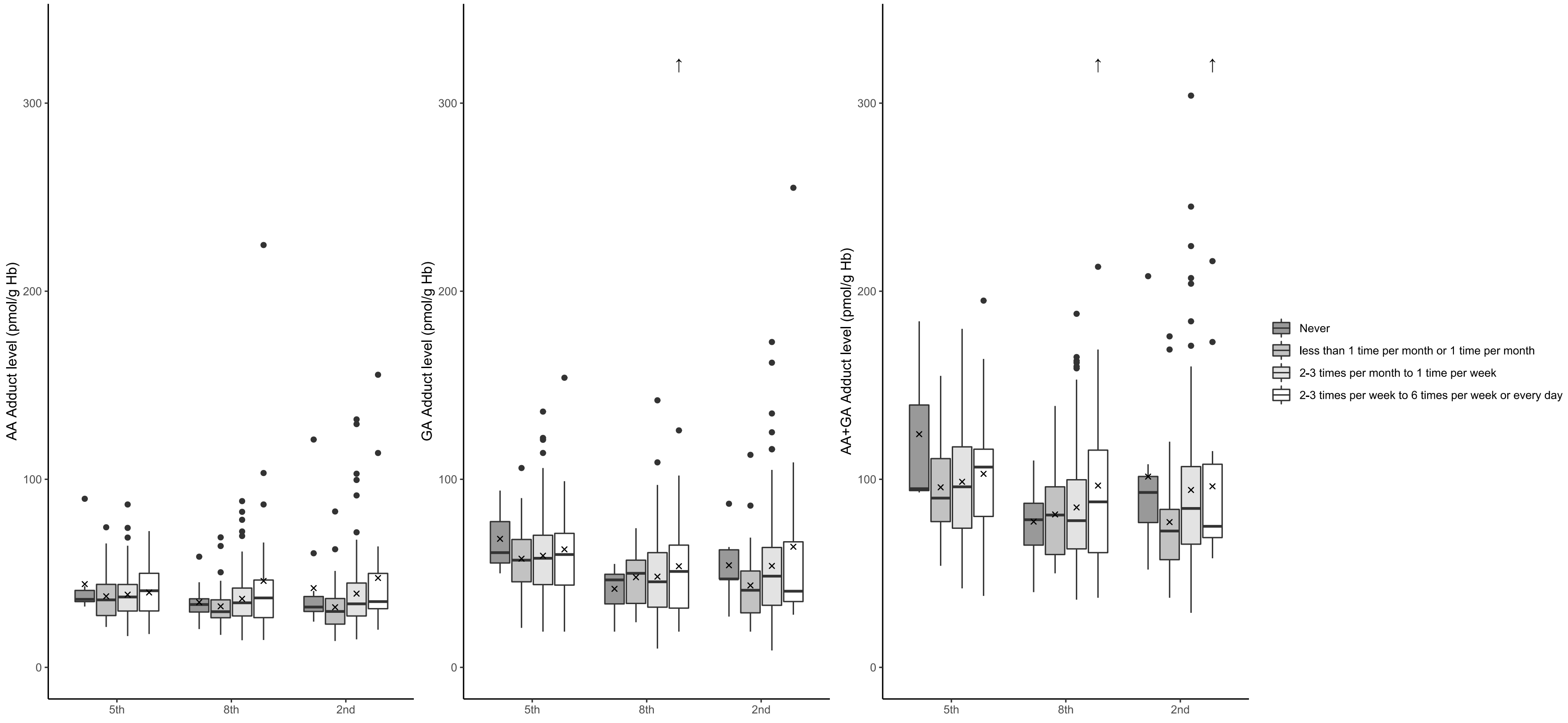

Supplementary Figure 5. ***Hb adduct levels in adolescents with different consumption frequencies of fried potatoes and french fries.*** *The level of Hb adducts of acrylamide (AA), glycidamide (GA), and the sum of acrylamide and glycidamide (AA+GA) is described as the mean (×), median (−), min/max and the quartiles in relation to different fried potatoes consumption frequencies for students in 5th grade (12 years), 8th grade (15 years) and 2nd grade of high school (18 years). The box denotes the interquartile range (IQR), with the lower quartile (Q1, 25th percentile), the median which is the second quartile (Q2, 50th percentile) and the upper quartile (Q3, 75th percentile). The whiskers show the range of ± 1.5 times the IQR. The values beyond the range of the whiskers are outliers and are marked with ●. The mean is denoted by ×, and outlier values over 320 pmol/g Hb are shown as ↑. Significant results in the Dunn's post hoc pairwise comparisons with Bonferroni correction are denoted with stars ($P \leq 0.05$ with *, $P \leq 0.01$ with **, and $P \leq 0.001$ with ***).*

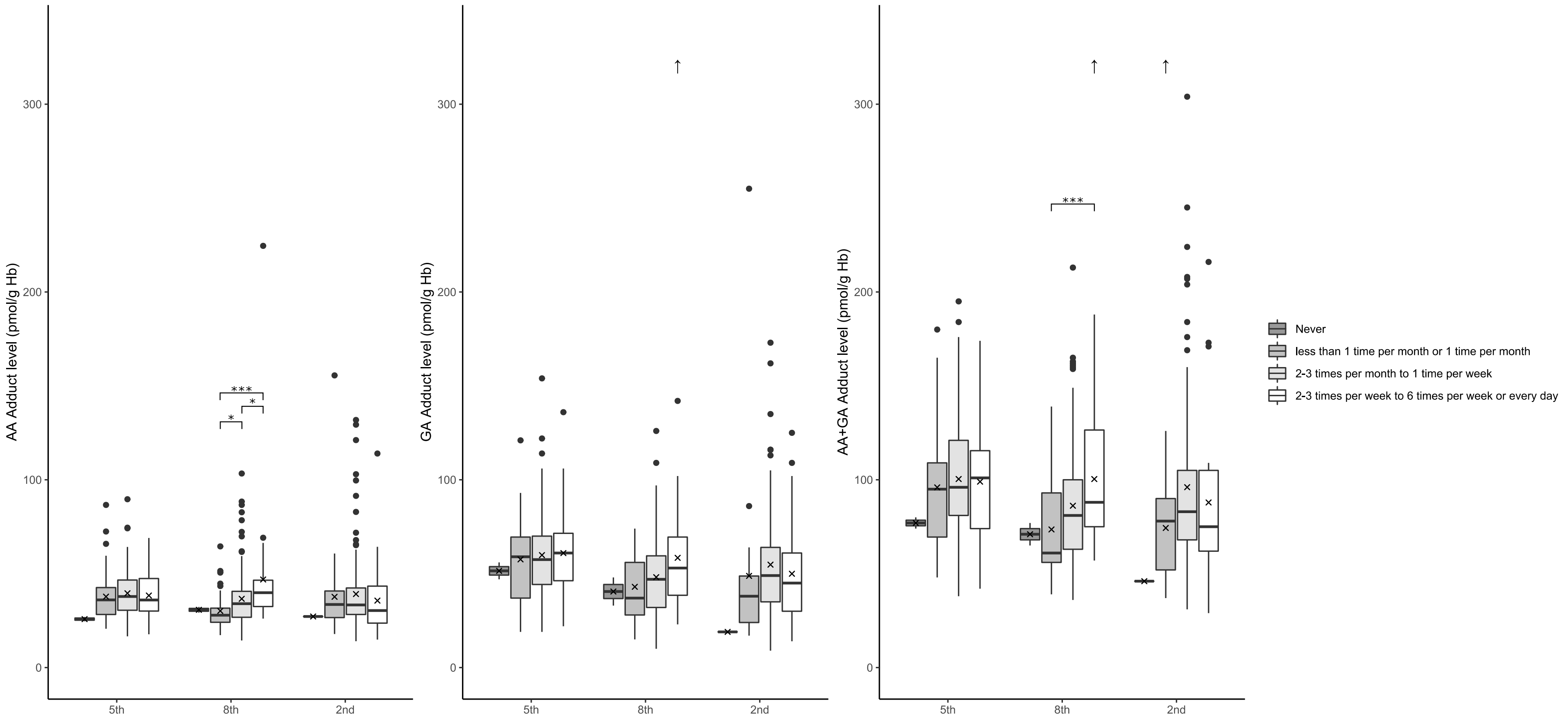

Supplement: Supplementary file 1 [file Data_Sheet_1.PDF]
